# Mechanosensitivity of Murine Lung Slowly Adapting Receptors: Minimal Impact of Chemosensory, Serotonergic, and Purinergic Signaling

**DOI:** 10.3389/fphys.2022.833665

**Published:** 2022-02-16

**Authors:** Nicolle J. Domnik, Sandra G. Vincent, John T. Fisher

**Affiliations:** ^1^Department of Biomedical and Molecular Sciences, Queen’s University, Kingston, ON, Canada; ^2^Department of Physiology and Pharmacology, Schulich School of Medicine and Dentistry, Western University, London, ON, Canada

**Keywords:** slowly adapting pulmonary receptor, murine (mouse), mechanosensation, chemosensation, purinergic, serotonergic

## Abstract

Murine slowly adapting receptors (SARs) within airway smooth muscle provide volume-related feedback; however, their mechanosensitivity and morphology are incompletely characterized. We explored two aspects of SAR physiology: their inherent static mechanosensitivity and a potential link to pulmonary neuroepithelial bodies (NEBs). SAR mechanosensitivity displays a rate sensitivity linked to speed of inflation; however, to what extent static SAR mechanosensitivity is tuned for the very rapid breathing frequency (B*_f_*) of small mammals (e.g., mouse) is unclear. NEB-associated, morphologically described smooth muscle-associated receptors (SMARs) may be a structural analog for functionally characterized SARs, suggesting functional linkages between SARs and NEBs. We addressed the hypotheses that: (1) rapid murine B*_f_* is associated with enhanced *in vivo* SAR static sensitivity; (2) if SARs and NEBs are functionally linked, stimuli reported to impact NEB function would alter SAR mechanosensitivity. We measured SAR action potential discharge frequency (AP *f*, action potentials/s) during quasi-static inflation [0–20 cmH_2_O trans-respiratory pressure (P_TR_)] in NEB-relevant conditions of hypoxia (F_I_O_2_ = 0.1), hypercarbia (F_I_CO_2_ = 0.1), and pharmacologic intervention (serotonergic 5-HT_3_ receptor antagonist, Tropisetron, 4.5 mg/kg; P2 purinergic receptor antagonist, Suramin, 50 mg/kg). In all protocols, we obtained: (1) AP *f* vs. P_TR_; (2) P_TR_ threshold; and (3) AP *f* onset at P_TR_ threshold. The murine AP *f* vs. P_TR_ response comprises high AP *f* (average maximum AP *f*: 236.1 ± 11.1 AP/s at 20 cmH_2_O), a low P_TR_ threshold (mean 2.0 ± 0.1 cmH_2_O), and a plateau in AP *f* between 15 and 20 cmH_2_O. Murine SAR mechanosensitivity (AP *f* vs. P_TR_) is up to 60% greater than that reported for larger mammals. Even the maximum difference between intervention and control conditions was minimally impacted by NEB-related alterations: Tropisetron −7.6 ± 1.8% (*p* = 0.005); Suramin −10.6 ± 1.5% (*p* = 0.01); hypoxia +9.3 ± 1.9% (*p* < 0.001); and hypercarbia −6.2 ± 0.9% (*p* < 0.001). We conclude that the high sensitivity of murine SARs to inflation provides enhanced resolution of operating lung volume, which is aligned with the rapid B*_f_* of the mouse. We found minimal evidence supporting a functional link between SARs and NEBs and speculate that the <10% change in SAR mechanosensitivity during altered NEB-related stimuli is not consistent with a meaningful physiologic role.

## Introduction

Several laboratories are known for seminal studies of the electrophysiologic characteristics of three primary populations of mammalian pulmonary vagal afferent fibers: myelinated, mechanosensitive slowly adapting (SARs) and rapidly adapting receptors (RARs; Adrian, 1933; Widdicombe and Nadel, 1963; Miserocchi and Sant’Ambrogio, 1974; Bartlett and Sant’Ambrogio, 1976; Sant’Ambrogio and Mortola, 1977), and non-myelinated, nociceptive C-fibers (Paintal, 1969; Coleridge and Coleridge, 1984; Lee and Pisarri, 2001; Carr and Undem, 2003; Canning et al., 2006). SARs are identified on the basis of an augmenting action potential (AP) discharge frequency (AP *f*, i.e., AP/s relative to airway pressure or trans-respiratory pressure, P_TR_) during inflation and a sustained discharge with minimal adaptation during maintained inflation [i.e., the adaptation index (Sant’Ambrogio et al., 1983; Coleridge and Coleridge, 1986; Widdicombe, 2009]. Despite a strong contemporary reliance on murine models in cardiopulmonary research, the vast majority of studies investigating SAR mechanoreceptor function have been performed in larger species (e.g., Widdicombe, 1954a; Mustafa and Purves, 1972; Miserocchi et al., 1973). Burnet and Hilaire addressed the murine SAR discharge profile with respect to tidal volume (Burnet and Hilaire, 1999); however, inflation volume does not address the fundamental link to the mechanoreceptor stimulus of AW pressure (Sant’Ambrogio, 1982, 1987; Sant’Ambrogio et al., 1984). More recently, Zhang et al. (2006) reported on murine respiratory reflexes, including recordings of SAR activity. We addressed the fundamental mechanosensitive properties of murine SARs referenced to AW pressure with a range of indices including static sensitivity (AP *f*) of the receptor to transmural pressure, P_TR_ threshold for activation, and the threshold AP *f*. We speculate that the rapid breathing frequency (B*_f_*) of the mouse, which ranges from approximately 150 to 200 breaths per minute at rest (Mortola and Noworaj, 1985; Vincent et al., 2007), would benefit from an enhanced static SAR mechanosensitivity in order to achieve sensory feedback on operating lung volumes (Vinegar et al., 1979). Feedback on operating lung volumes is important in health and disease (O’Donnell et al., 2016, 2017), as well as in breath-by-breath regulation of B*_f_* and tidal volume.

SARs have been suggested to be linked to a morphologically identified subset of lung afferents with morphologically identified connections to neuroepithelial bodies (NEBs; Brouns et al., 2000, 2006a; Adriaensen et al., 2006), although exploration of the physiologic implications of such links remain unexplored. NEBs are clusters of pulmonary neuroendocrine cells (PNECs) located exclusively within the airway epithelium (Lauweryns and Cokelaere, 1973; Linnoila, 2006; Domnik and Cutz, 2011; Cutz et al., 2013) and are heavily innervated by various nerve populations, primarily comprised of vagal afferent fibers (Brouns et al., 2006a). While investigations into NEB morphology and function were historically carried out in rodent models, more recently, whole-mount characterization of airway innervation has confirmed innervated PNEC clusters in humans (West et al., 2015). Traditional characterization of the neural populations innervating NEBs relied heavily on histologic and immunohistochemical approaches; however, molecular and genetic techniques have revealed a purinergic receptor-expressing vagal afferent population innervating PNECs and mediating traditional reflex apneic responses vis-a-vis the mechanosensitive cation channel Piezo2 (Chang et al., 2015; Nonomura et al., 2017).

Adriaensen and colleagues advanced the hypothesis that NEBs play a role in *in vivo* pulmonary mechanosensation (Adriaensen et al., 2006; Lembrechts et al., 2011, 2012). This is supported by the morphological identification of Na^+^/K^+^-ATPase on a subpopulation of NEB-innervating vagal fibers (Adriaensen et al., 2006; Brouns et al., 2006a) and NEB expression of the 2-pore K^+^ channel, TRAAK (Lembrechts et al., 2011). Expression of Na^+^/K^+^-ATPase on a population of vagal afferent free nerve terminals innervating the airway smooth muscle (ASM), described as “smooth muscle-associated receptors” (SMARs) and potentially a morphologic counterpart of electrophysiologically characterized SARs, led Adriaensen et al. to propose that NEB-innervating vagal fibers and SMARs/SARs may be functionally linked (Adriaensen et al., 2006; Brouns et al., 2006a,b). This remains an area of ongoing discussion: in electrophysiologic studies on isolated SARs, where punctate stimulation was used to identify and dissect the SAR receptive field, subsequent staining and examination of the receptive field tissue has not yielded NEBs within these histological sections (Yu et al., 2004). Neural tracing experiments originating in the nodose ganglion reported differing sensory end-structures of NEBs and SARs (Yu, 2007). Furthermore, it has been argued that since SARs are neither SP-immunoreactive or CGRP-immunoreactive, it is unlikely that NEBs are connected to mechanosensors (Lee and Yu, 2014). Thus, the relationship between these entities remains undefined: if NEBs participate in the mechanotransduction of SARs, this may be in a direct or modulatory role.

We reasoned that if SARs are functionally linked to NEBs, factors influencing NEB responsiveness should reasonably be expected to also impact SAR activity. NEBs were initially identified as hypoxia sensors based on synaptic release of serotonin (5-HT) during hypoxia exposure (Lauweryns and Cokelaere, 1973; Lauweryns et al., 1977; Fu et al., 2001; Pan et al., 2006) and the detection of hypoxia by a membrane-delimited O_2_ sensor, NADPH oxidase 2 (NOX2), coupled with a hypoxia-sensitive K^+^ channel, Kv3.3a (Youngson et al., 1993; Wang et al., 1996; Fu et al., 2000; O’Kelly et al., 2001). Analogous to the polymodal, peripheral chemosensor, and the carotid body, NEBs have since been described as multimodal airway sensors based on their responsiveness to hypoxia, as well as to hypercarbia/acidosis and mechanical stretch *in vitro* (Lembrechts et al., 2012; Livermore et al., 2015).

Two mediators are proposed as the primary means of NEB signaling to adjacent cells: serotonin (5-hydroxytryptamine; 5-HT) and ATP (De Proost et al., 2008). The 5-HT type 3 receptor (5-HT_3_R), which is thought to have an auto-excitatory role, is expressed by PNECs/NEBs (Fu et al., 2001), on nodose-derived vagal fibers in the rat (Rosenberg et al., 1997), and on dissected cat and rabbit vagal fibers (Hoyer et al., 1989). NEB-innervating vagal fibers have not yet been assessed for the presence of 5-HT_3_R. In addition to 5-HT_3_R, NEBs express purinergic P2X_2_ and P2X_3_ receptors (Fu et al., 2004) and produce significant quantities of ATP. ATP release is implicated in cell-to-cell signaling between NEB cells (ATP-releasing) and P2Y_2_-expressing club-like cells (De Proost et al., 2008). P2X_2_R and P2X_3_R have also been directly identified on vagal afferent fibers innervating NEBs in the mouse (Brouns et al., 2000, 2006a, 2009). Select studies have demonstrated direct *in vitro* or *in vivo* activation of mechanosensitive vagal afferents by either ATP or 5-HT (Mazzone and Undem, 2016). For example, the direct activation of SAR, RAR, and C-fibers by ATP (Pelleg and Hurt, 1996; Canning et al., 2004; Undem et al., 2004; Kollarik and Undem, 2006) and the direct activation of RAR and C-fibers by 5-HT (Canning et al., 2006). Thus, it has been proposed that NEBs may initiate or modulate vagal afferent function through release of ATP or 5-HT (Lembrechts et al., 2012; Adriaensen et al., 2015).

We tested two hypotheses concerning the *in vivo* physiology of murine SAR function: (1) that the rapid B*_f_* of mice is associated with an enhanced *in vivo* SAR mechanosensitivity, evidenced through a low threshold of P_TR_ activation, and high AP *f* static P_TR_ sensitivity profile and (2) that exposure to NEB-related stimuli (hypoxia, hypercarbia, and pharmacologic blockade of 5-HT_3_R and P2R) would alter SAR mechanosensitivity *via* putative functional links between SARs and NEBs. To test our hypotheses, we measured electrophysiologic single-fiber SAR afferent discharge during slow, quasi-static inflation from 0 to 20 cmH_2_O of P_TR_ in the mouse and assessed the impact of multiple interventions known to impact NEB physiology.

## Materials and Methods

All protocols met the Canadian Council of Animal Care standards and were approved by the Queen’s University Animal Care Committee.

### Surgical Preparation

**Figure 1:**
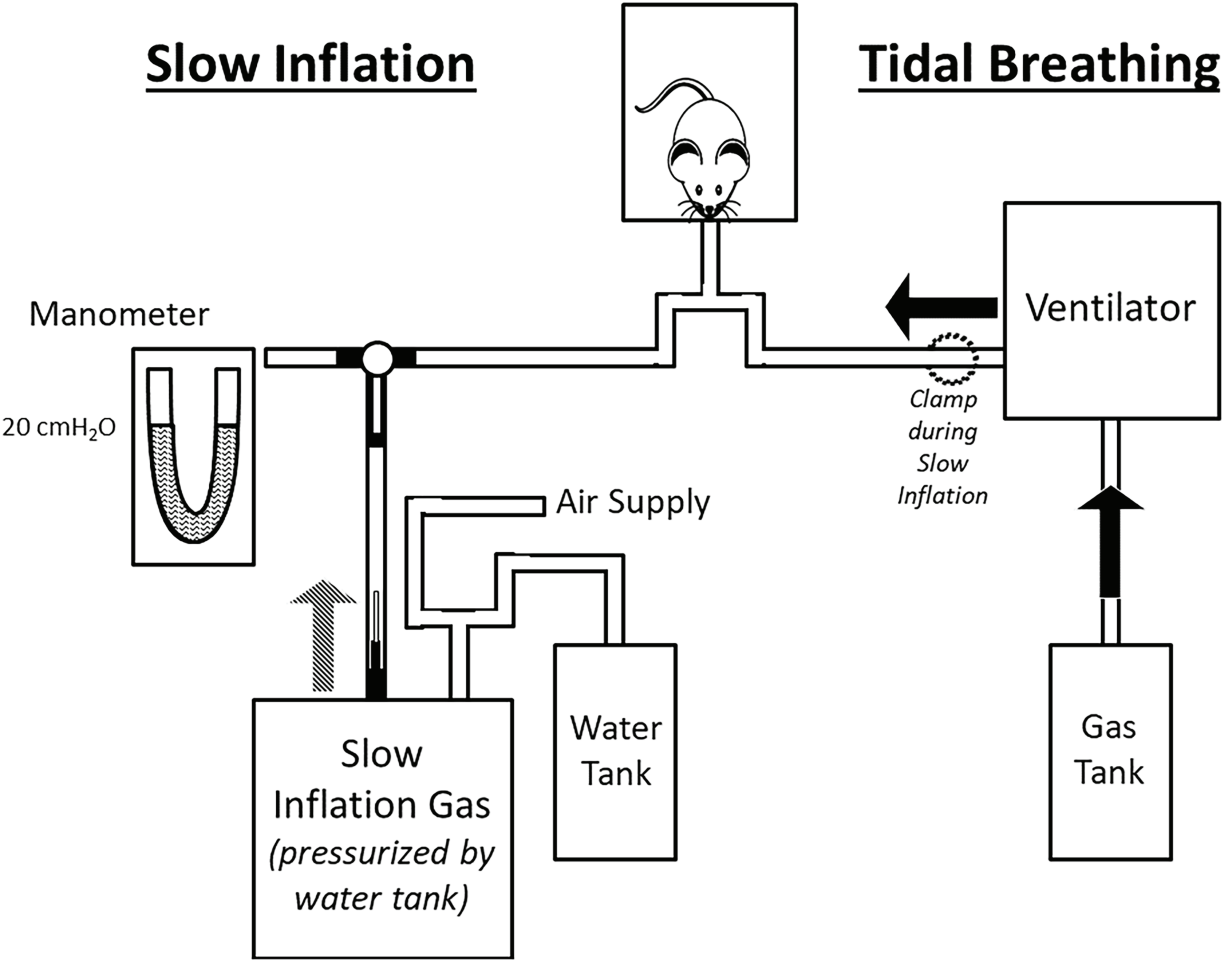
Schematic of experimental set-up (not to scale). The experimental set-up comprised two circuits: one used during tidal breathing **(right side)** and one for slow inflation **(left side)**. *Tidal Breathing*: ventilation was achieved *via* a volume-ventilator supplied with experimental gas mixture. The slow inflation components were excluded from the circuit during tidal breathing by closure of a 3-way stopcock (see black T-shaped connection between manometer/slow inflation gas/animal). *Slow Inflation*: During slow inflation, the ventilator was turned off and the line to the animal clamped. Rotation of the stopcock connected the slow inflation system to the animal. The lungs were inflated slowly to the desired pressure set by the drum of compressed air, which was calibrated *via* manometer prior to the trial.

**Figure 2:**
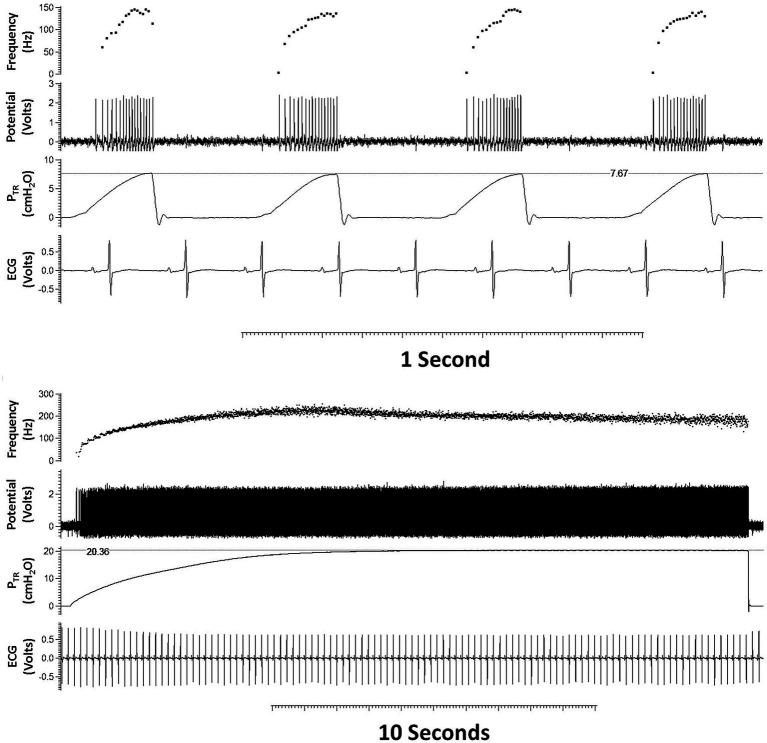
Typical tracing of a single slowly adapting receptor (SAR) during tidal breathing **(top panel)** and quasi-static inflation to 20 cmH_2_O **(bottom panel)**. From top to bottom within each panel are shown: AP *f* (AP/s), action potentials [Potential (Volts)], trans-respiratory pressure (P_TR_), and heart rate [ECG (Volts)]. The SAR demonstrates characteristic augmenting discharge frequency during tidal inflation **(upper panel)** and sustained discharge frequency at a maintained pressure (bottom tracing).

Male adult C57BL/6 mice were anaesthetized using sodium pentobarbital (60 mg/kg, 30 mg/ml; Ceva Santé Animal, Libourne, France) and, following tracheal cannulation, were mechanically ventilated (120 breaths/min; Harvard Rodent Ventilator Model 683, Harvard Apparatus, South Natick, MA, United States; Figure 1). The mouse was placed on a heating pad set to 37° to maintain body temperature. The left jugular vein was catheterized for *intravenous* administration of maintenance anesthetic and pharmacologic agents. The paralyzing agent, pancuronium bromide (0.25 mg/kg), was administered prior to hypercarbia and hypoxia trials to eliminate respiratory efforts. The left cervical vagus nerve was isolated and transected high in the neck. Following de-sheathing of the nerve, filaments were viewed under a surgical microscope and dissected from the peripheral cut-end using watchmaker forceps and iridectomy scissors. The nerve was constantly immersed in mineral oil to prevent drying out and provide electrical isolation. Action potentials were recorded *via* extracellular, bipolar platinum electrode connected to a head stage and amplifier, and P_TR_ and ECG were also recorded (AP: AI 401 Amplifier and CyberAmp Smart Probe, sampling frequency = 20,000 samples/s; P_TR_: Motorola pressure transducer, AI 490 amplifier, CyberAmp 380 Signal Conditioner, sampling frequency = 1,000 samples/s; ECG: A M Systems Model 1700 differential AC amplifier, sampling frequency = 1,000 samples/s). Recordings from single SARs obtained from isolated nerve filaments were identified based on their AP *f* pattern and adaptation to a maintained inflation using waveform recognition in Spike 2 (software Version 7.02, Cambridge Electronic Design Ltd., Cambridge, United Kingdom; Figure 2).

### Experimental Set-Up

A schematic of the experimental set-up is provided in Figure 1. Tidal breathing was achieved by mechanical ventilation at 120 breaths/min, with end-expiratory P_TR_ = 0 cmH_2_O and end-inspiratory P_TR_ during tidal breathing ~7–8 cmH_2_O. Due to the high chest wall compliance of the mouse, P_TR_ is a close surrogate for transpulmonary pressure. Therefore, we maintained the chest wall intact throughout. Slow, quasi-static inflations were achieved by stopping mechanical ventilation and switching the inspiratory line to a 10 L drum pressurized to 20 cmH_2_O. Resistance from a 27-gage needle within the tubing ensured a slow inflation of ~2 cmH_2_O/s. Mechanical ventilation was resumed upon completion of the quasi-static inflation.

### Protocol: Naïve vs. Chemosensory vs. Pharmacologic Recordings

Naïve baseline recordings consisted of two hyperinflations of the lung to total lung capacity (TLC) to establish a constant volume history (Bernstein, 1957; Mead and Collier, 1959) followed by a period of tidal breathing (90 s) preceding quasi-static inflation from 0 to 20 cmH_2_O over ~10 s. Target pressure (20 cmH_2_O) was then maintained for an additional 10 s before tidal breathing was resumed for either 90 s or 3 min (for the chemosensory vs. pharmacologic challenges, respectively). Naïve recordings were conducted with a ventilatory mixture of 40% O_2_, 60% N_2_ to maintain hemoglobin O_2_ saturation.

Chemosensory challenges were similarly preceded by two volume history inflations but followed by a 90 s ventilation period with the test gas (hypoxia: F_I_O_2_ = 0.1, i.e., 10% O_2_ in 90% N_2_; hypercarbia: F_I_CO_2_ = 0.1, i.e., 10% CO_2_ in 90% O_2_) and then a quasi-static inflation as per the naïve protocol. This was followed by an additional 60 s of tidal breathing with the test gas, followed by 60 s of ventilation with the naïve gas concentration. Preliminary oximetry experiments determined the time course for desaturation during hypoxic ventilation. Mice displayed 98% saturation at baseline (FIO_2_ = 0.4) vs. 35–40% saturation within 60 s of hypoxic (FIO_2_ = 0.1) ventilation (MouseOx, STARR Life Sciences Corp., Oakmont, PA, United States; data not shown).

**Figure 3:**
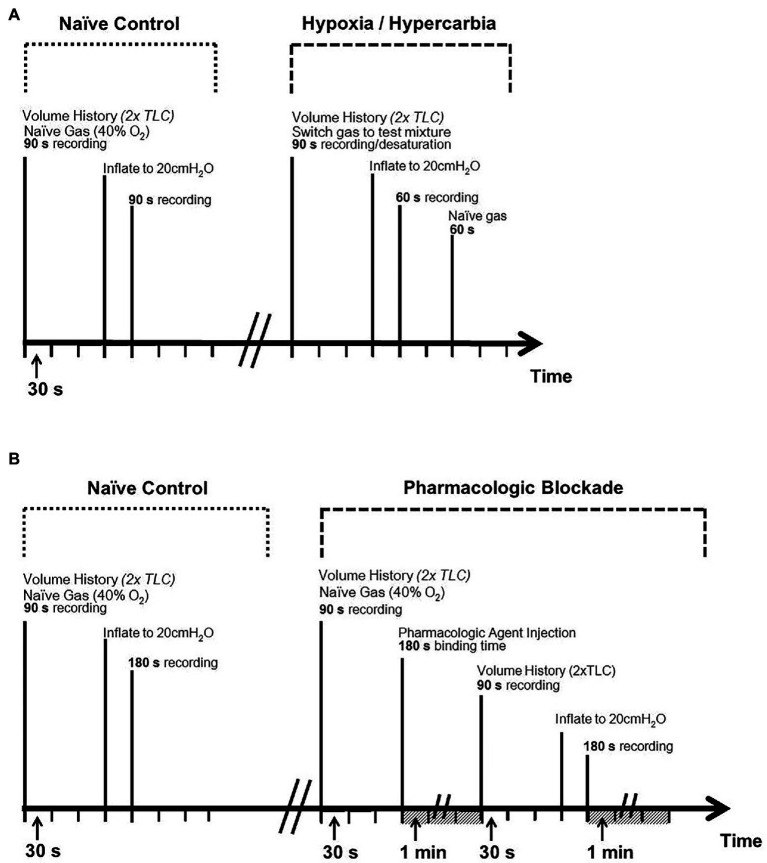
Experimental protocol timelines. Top panel **(A)**: timeline for chemosensory challenges (hypoxia and hypercarbia). Bottom panel **(B)**: timeline for pharmacologic challenges [serotonergic (Tropisetron) and purinergic (Suramin) blockade]. Volume history consisted of two hyperinflations to total lung capacity (TLC) prior to experiment to ensure standardized pulmonary compliance between trials.

Pharmacologic challenges consisted of a 90 s baseline of tidal breathing, followed by *intravenous* drug administration of either an antagonist of serotonergic 5-HT_3_ receptors, Tropisetron (Tropisetron hydrochloride, 4.5 mg/kg, Tocris Bioscience, Bristol, United Kingdom), or an antagonist of P2 purinergic receptors, Suramin (Suramin hexasodium salt, 50 mg/kg, Tocris Bioscience, Ellisville, MO, United States), 3 min of tidal ventilation prior to two volume history inflations, and 90 s of recorded tidal breathing (akin to the naïve protocol). This was followed by the quasi-static inflation to 20 cmH_2_O and a final 3-min tidal breathing period. Both Suramin (De Proost et al., 2009) and Tropisetron (Fu et al., 2001, 2002) have previously been successfully used in *ex vivo* investigation of NEB function or the NEB microenvironment. An *in vivo* Suramin dose of 50 mg/kg was chosen herein for its efficacy in the absence of significant toxic or central effects (Spigelman et al., 1987; Bouteille et al., 1995; Russell et al., 2001; Kharlamov et al., 2002; Joseph et al., 2004), while an *in vivo* Tropisetron dose of 4.5 mg/kg was similarly chosen for its efficacy while avoiding the partial agonism of nicotinic α7 receptors observed at higher doses (Seynaeve et al., 1991; Hashimoto et al., 2006; Aminian et al., 2013; Zirak et al., 2014, 2020; Amiri et al., 2015; Hosseinzadeh et al., 2021). Full protocol timelines are given in Figure 3 and a summary of the number of animals as well as individual afferent fibers analyzed (i.e., *n* per protocol) is provided in Table 1.

**Table 1:** Summary of experiments, indicating number of animals, and individual afferent fibers analyzed.

Protocol		Animals (*n* mice)	Nerve Fibers (*n* fibers)
Chemosensory	*Hypoxia*	14	28
	*Hypercarbia*	10	40
Pharmacologic	*Tropisetron*	11 (7)^*^	11 (8)*
	*Suramin*	6	7

Nerve fiber *n* > animal *n* when multiple fibers could be clearly identified and analyzed within a single recording from one animal or when multiple recordings from different single fibers could be performed in series in one animal during chemosensory challenges.

* *n* = 11 refers to the number of mice and fibers used in baseline SAR characterization; *n* = 7 or *n* = 8 (in brackets) indicates the n used in comparisons between naïve and Tropisetron challenge.

### Analysis

Data files were exported into Excel from Spike 2. Mice exhibiting tonic baseline activity (defined as a regular AP *f* at functional residual capacity, FRC, where P_TR_ = 0 cmH_2_O) were not included in the calculation of P_TR_ threshold or first firing frequency. Statistical analyses were performed in SigmaPlot 13 (Systat Software Inc., San Jose, CA, United States) and consisted of 2-way ANOVA with repeated measures (parametric, ranked testing when necessary), paired *T*-tests, linear regression, and post-hoc analysis where appropriate. Tests performed are indicated alongside Results. Statistical significance was set to *p* < 0.05. All data are mean ± SEM.

## Results

**Figure 4:**
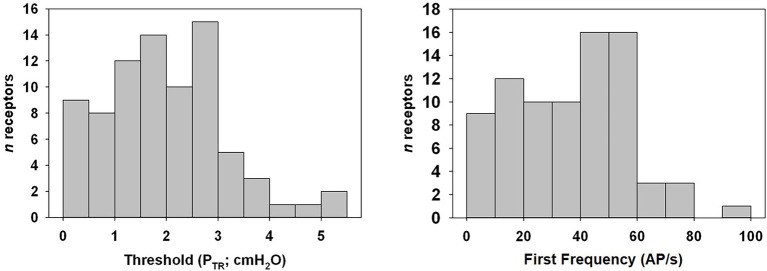
Distribution histogram of P_TR_ threshold of activation and first frequency of murine SARs. Tonic receptors, active at FRC or 0 cmH_2_O (*n* = 5), were excluded from analysis. Note that the majority of SARs was active at relatively low pressures.

**Figure 5:**
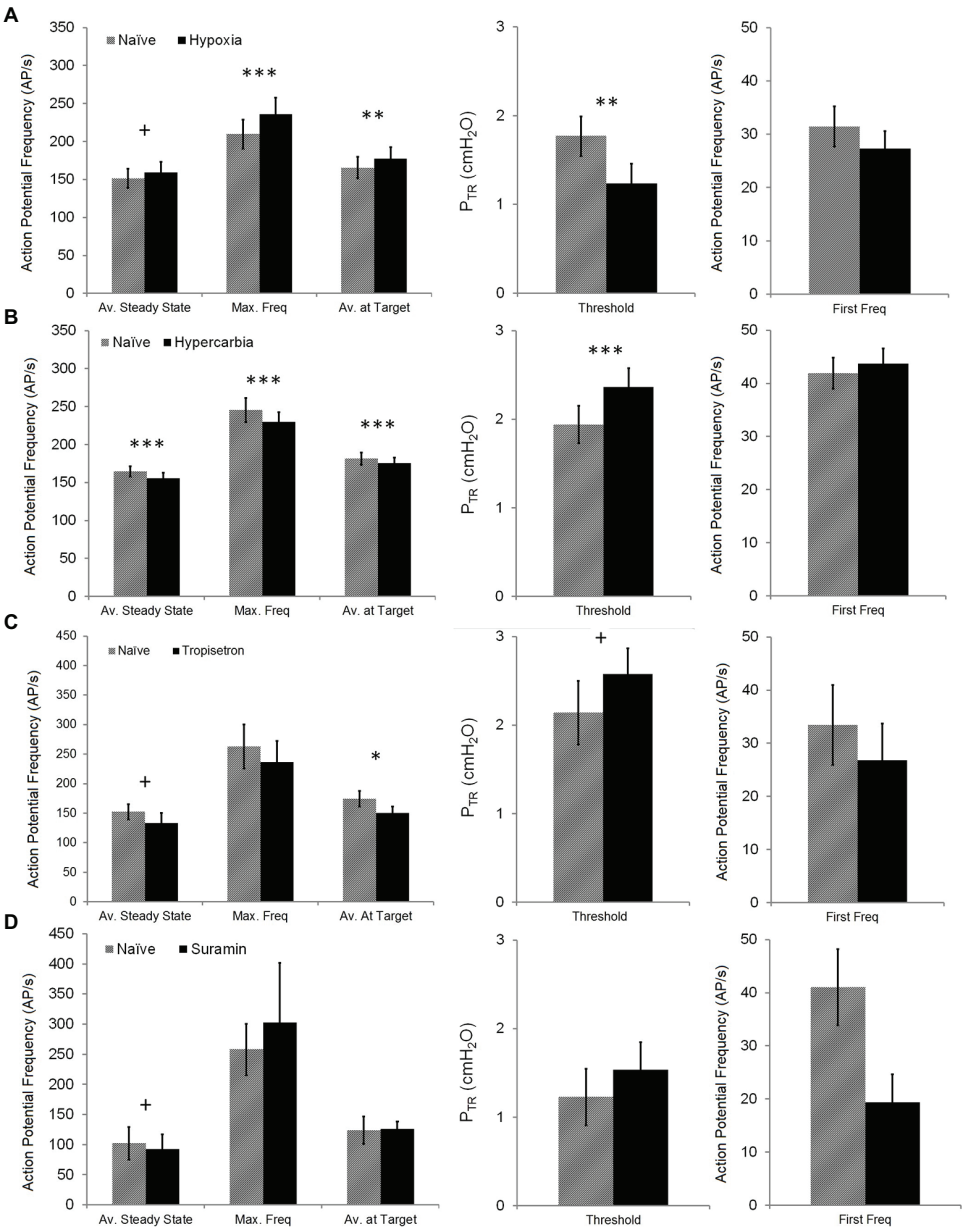
Impact of chemosensory or pharmacologic challenges on SAR activation and steady state SAR activity. The impact of hypoxia (top row—**A**), hypercarbia (second row—**B**), Tropisetron (third row—**C**), and Suramin (bottom row—**D**) on Av. Steady State (mean AP *f* over 1 s, measured 5 s after target P_TR_ of 20 cmH_2_O was attained), Max. Freq (maximum single frequency), Av. at Target (mean AP *f* over 1 s measured when target P_TR_ of 20 cmH_2_O was attained), Threshold (P_TR_ pressure required to initiate SAR activation during slow inflation), and First Freq (instantaneous AP *f* at SAR activation onset) was assessed. Hypoxia had an opposite effect on Av. Steady State (increased) compared with hypercarbia, Tropisetron, and Suramin (decreased). Hypoxia also increased Max. Freq and Av. at Target, whereas these were decreased in hypercarbia (Av. at Target was also decreased in Tropisetron). Conversely, hypoxia decreased Threshold, while hypercarbia and Tropisetron increased Threshold. There was no impact of any intervention on first firing frequency. Difference between naïve and test conditions: + *p* < 0.05; *p < 0.01; **p = 0.001; and ***p < 0.001. All data are mean ± SEM.

**Figure 6:**
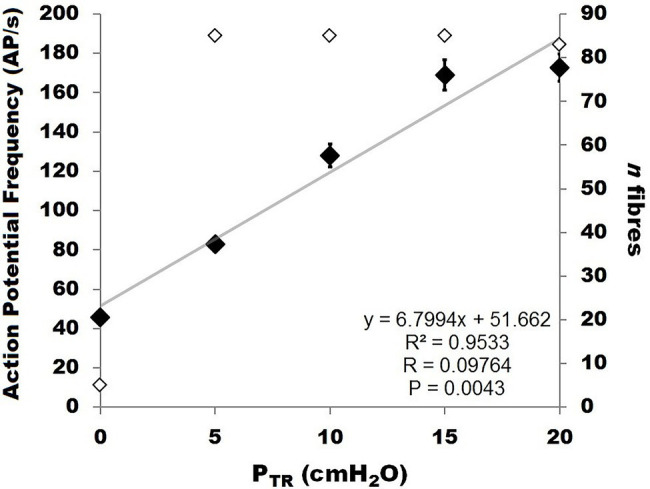
Relationship between murine SAR action potential frequency (AP *f*; labeled “action potential frequency”; **left** Y-axis, black diamonds) and pressure (P_TR_) during slow inflation from 0 to 20 cmH_2_O. The data were best fit by a linear correlation between P_TR_ and AP *f* (*R*^2^ = 0.9533, *p* < 0.01). The number of receptors contributing to AP *f* at each pressure value is indicated on the **right** Y-axis (open diamonds).

**Figure 7:**
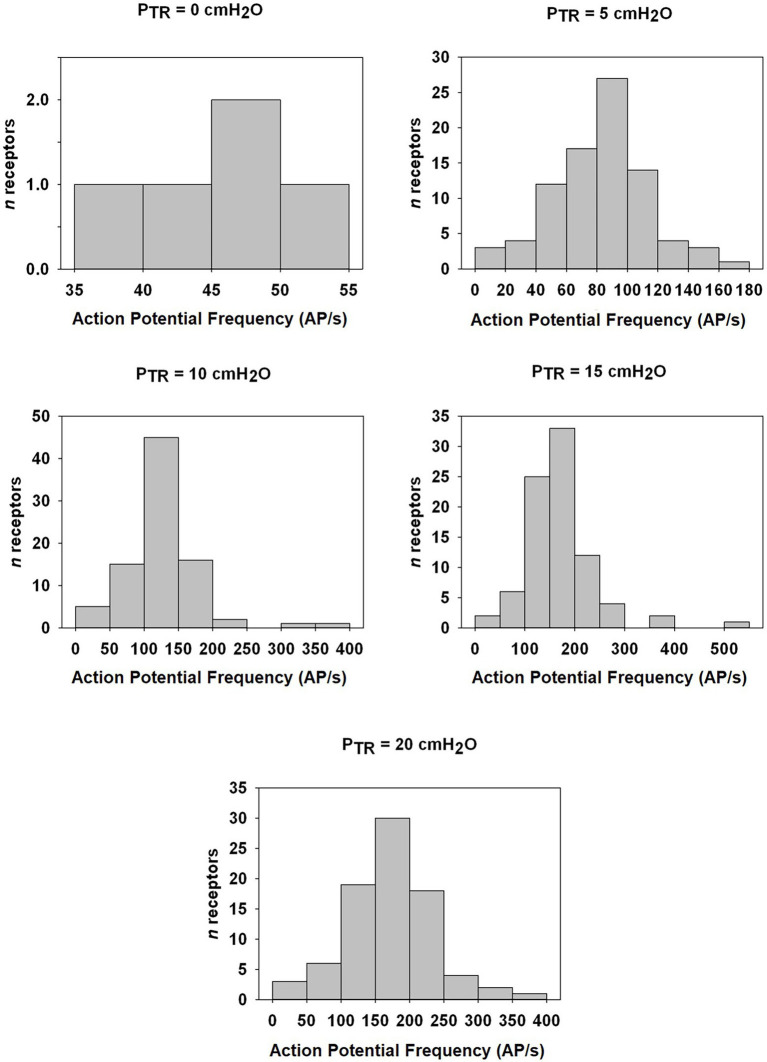
Distribution histogram of Action Potential frequency (AP *f*) among murine SARs at indicated P_TR_ from FRC (0 cmH_2_O) to TLC (20 cmH_2_O). The number of fibers and the AP *f* in AP/s is indicated for static pressures of 0, 5, 10, 15, and 20 cmH_2_O. Note the change in Y-axis ranges between graphs.

**Figure 8:**
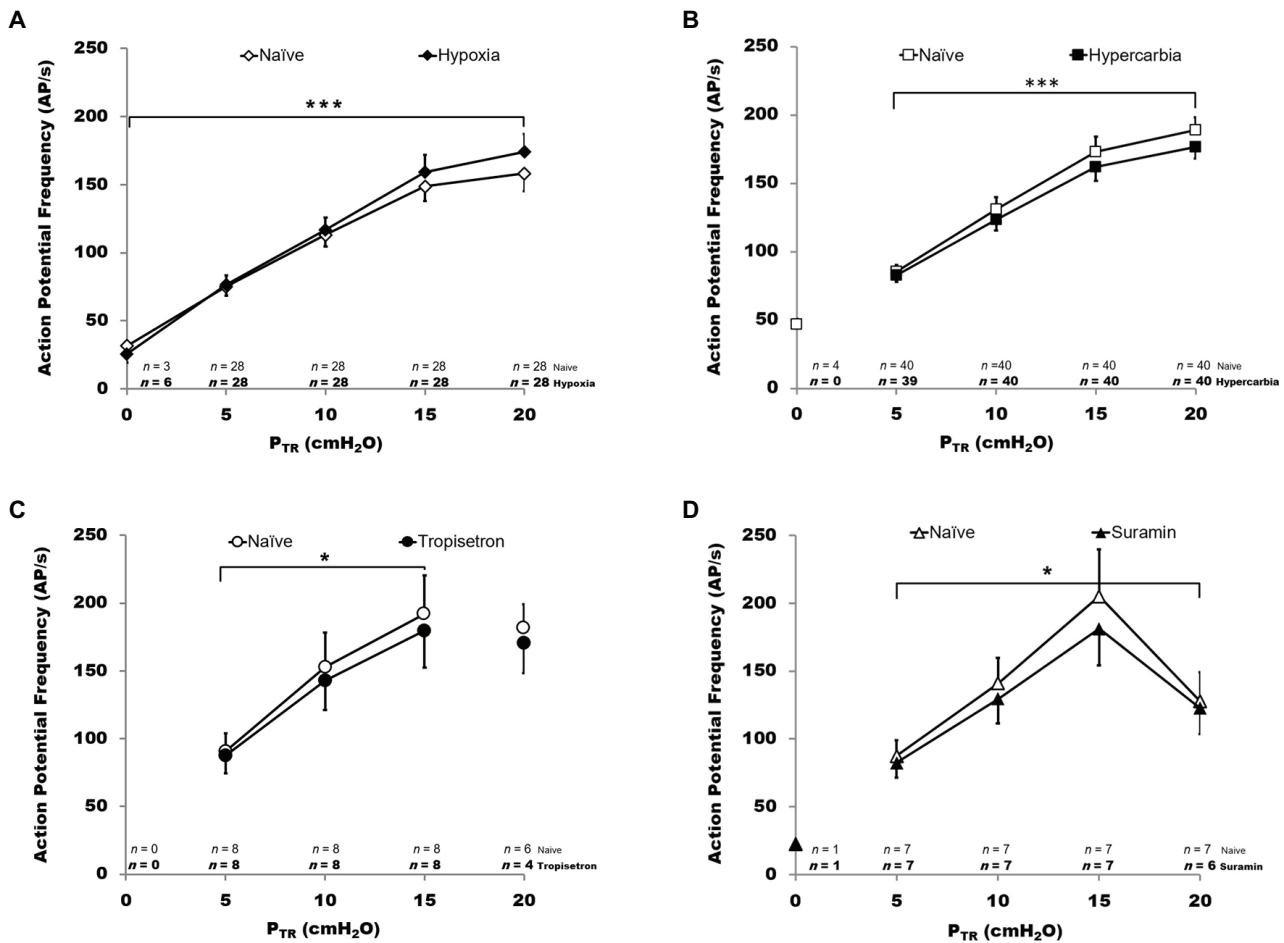
Impact of chemosensory and pharmacologic challenge on SAR activity during slow inflation. Action potential frequency (AP *f* in AP/s) is plotted for P_TR_ from 0 to 20 cmH_2_O. In all graphs, hollow symbols represent the naïve trials and filled symbols represent the experimental condition. The *n* values at the bottom of each panel indicate the number of SARs active (*n*) at each pressure, with experimental condition *n* indicated in bold below the naïve condition *n* values. *Hypoxia* (*panel **A**, top left*): hypoxia was associated with a small but statistically significant increase in AP *f* (*p* < 0.001). *Hypercarbia* (*panel **B**, top right*): in contrast, hypercarbia was associated with a small but statistically significant decrease in AP *f* (*p* < 0.001). *Tropisetron* (*panel **C**, bottom left*): pretreatment with Tropisetron was associated with a small but statistically significant decrease in AP *f* (*p* < 0.01). *Suramin* (*panel **D**, bottom right*): pretreatment with Suramin was associated with a small but statistically significant decrease in AP *f* (*p* < 0.01). All data are mean ± SEM.

Two aspects of SAR behavior were assessed before (naïve) and after chemosensory/pharmacologic challenge: (i) behavior at the pressure threshold of activation (i.e., activation P_TR_ and first firing frequency; Figures 4, 5) and (ii) their AP *f* throughout inflation to, and during sustained inflation at, P_TR_ = 20 cmH_2_O (Figures 6–8).

### Intrinsic SAR Behavior (Naïve Protocol)

Of the 85 naïve SARs analyzed, five (5.9%) showed tonic activity at FRC (end-expiratory P_TR_ = 0 cmH_2_O) and were therefore excluded from the analysis of the P_TR_ threshold for activation (Threshold) or the first firing frequency. The latter was calculated from SARs displaying a clear onset of activity during inflation at P_TR_ > 0 cmH_2_O. The mean Threshold for SAR activation of the remaining 80 SARs was 2.0 ± 0.1 cmH_2_O, and the mean first firing frequency was 36.8 ± 2.2 AP/s (Figure 4: distribution histogram). The average of the maximum instantaneous frequencies recorded for each SAR was 236.1 ± 11.1 AP/s when considering all SARs (*n* = 85 including the five tonic SARs).

SAR discharge was examined throughout the course of the slow inflation, and mean AP *f* values calculated at 5, 10, 15, and 20 cmH_2_O. A linear correlation was found to have the strongest fit within these data, and there was a positive linear correlation between P_TR_ and AP *f* (*R* = 0.9764, *p* < 0.01; Figure 6). The slope represents a SAR population mechanosensitivity of approx. 6.8 AP/s per cmH_2_O, globally, when including all SARs (including the *n* = 5 active at FRC). While most SARs appeared to plateau between 15 and 20 cmH_2_O, AP *f* at these values varied significantly across the examined SARs (*p* < 0.001). The SAR distribution histogram of AP *f* at each pressure (0, 5, 10, 15, and 20 cmH_2_O) is illustrated in Figure 7. The mean AP *f* range of SAR activity from 45.9 ± 2.3 AP/s (at 0 cmH_2_O, i.e., tonically active SARs) to 172.8 ± 6.9 AP/s (at 20 cmH_2_O, all SARs active) represented a 376% increase in activity (i.e., change in mechanosensitivity of 18.8%/cmH_2_O) during inflation from FRC to TLC. When considering only those SARs that were not active at FRC (*n* = 80), the estimated SAR population mechanosensitivity is approximately 8.6 AP/s per cmH_2_O.

### SAR Behavior During Chemosensory or Pharmacologic Challenge: Onset and Maintenance

The impact of chemosensory or pharmacologic challenge was assessed by comparing Threshold for SAR activation, first firing frequency, maximum instantaneous frequency, and the average AP *f* at initial Target and Steady State (20 cmH_2_O) pressures between naïve and challenge values. Chemosensory experiments compared naïve trials (FIO_2_ = 0.4) with either hypoxia (FIO_2_ = 0.1) or hypercarbia (FICO_2_ = 0.1, FIO_2_ = 0.9). Hypoxia resulted in a small decrease in the P_TR_ threshold for SAR activation during slow inflation (naïve: 1.8 ± 0.2 cmH_2_O, hypoxia: 1.2 ± 0.2 cmH_2_O; *p* < 0.01 − Wilcoxon Signed Rank *T*-Test), whereas hypercarbia increased the P_TR_ threshold of SAR activation (naïve: 1.9 ± 0.2 cmH_2_O, hypercarbia: 2.4 ± 0.2 cmH_2_O; *p* < 0.001 − Paired *T*-Test; Figure 5). Pretreatment with Tropisetron caused an increase in the P_TR_ threshold for SAR activation (naïve: 2.1 ± 0.4 cmH_2_O, Tropisetron: 2.6 ± 0.3 cmH_2_O; *p* < 0.05 − Paired *T*-Test, Figure 5), but there was no effect of pretreatment with Suramin on the P_TR_ threshold for SAR activation. The first frequency at the P_TR_ threshold was not different during chemosensory or pharmacologic challenge.

The mean discharge at target pressure (20 cmH_2_O) was slightly increased (<10%) during hypoxic ventilation (naïve: 165.5 ± 14.1 AP/s, hypoxia: 177.2 ± 15.4 AP/s; *p* = 0.001 − Wilcoxon Signed Rank *T*-Test; Figure 5) and slightly reduced during hypercarbic ventilation (naïve: 181.4 ± 8.0 Hz, hypercarbia: 175.3 ± 7.6 AP/s; *p* < 0.001 − Paired *T*-Test) or pretreatment with Tropisetron (naïve: 174.3 ± 13.2 AP/s, Tropisetron: 150.3 ± 11.3; *p* < 0.01 − Paired *T*-Test, Figure 5). Pretreatment with Suramin did not alter mean discharge at target pressure. Similarly, mean discharge steady state AP *f* was increased during hypoxia (naïve: 151.2 ± 12.6 AP/s, hypoxia: 159.1 ± 13.8 Hz; *p* < 0.05 − Wilcoxon Signed Rank *T*-Test) and decreased during hypercarbia (164.7 ± 6.7 AP/s, hypercarbia: 155.9 ± 7.2 Hz; *p* < 0.001 − Wilcoxon Signed Rank *T*-Test) or after pretreatment with Tropisetron (naïve: 152.5 ± 12.7 AP/s, Tropisetron: 141.9 ± 15.3; *p* < 0.05 − Paired *T*-Test). Additionally, pretreatment with Suramin resulted in a statistically lower mean discharge steady state frequency (naïve: 102.0 ± 27.1, Suramin: 92.4 ± 24.5; *p* < 0.05 − Paired *T*-Test). Hypoxia and hypercarbia showed similar, but opposing effects on maximum instantaneous frequency, with hypoxia increasing (naïve: 209.7 ± 19.3, hypoxia: 235.9 ± 22.0; *p* < 0.001 − Wilcoxon Signed Rank *T*-Test) and hypercarbia decreasing maximum instantaneous frequency (naïve: 245.6 ± 16.0, hypercarbia: 229.9 ± 12.9; *p* < 0.001 − Wilcoxon Signed Rank *T*-Test). There was no effect of Tropisetron or Suramin on maximum instantaneous frequency.

### SAR Behavior During Chemosensory or Pharmacologic Challenge: Slow Inflation

SAR activity was analyzed during the slow inflation by comparing mean values as well as the percent change between naïve and test conditions. There was a significant effect of P_TR_ on AP *f* (*p* < 0.001), increased mean AP *f* in hypoxia (*p* < 0.001), and an interaction between naïve vs. hypoxic condition and pressure (*p* < 0.001, all Figure 8, 2-way RM ANOVA, ranked data). As only two receptors were active at P_TR_ = 0 cmH_2_O during the hypoxia trial, P_TR_ = 0 cmH_2_O was excluded from analysis. There was a significant effect of P_TR_ on AP *f* (*p* < 0.001) during hypercarbic exposure, decreased mean AP *f* in hypercarbia (*p* < 0.001), and an interactive effect of naïve vs. hypercarbic condition and pressure (*p* < 0.05; all Figure 8, 2-way RM ANOVA, ranked data). There were no fibers active at 0 cmH_2_O in the naïve vs. hypercarbia trials.

The impact of pretreatment with Tropisetron was analyzed over a pressure range of 5–15 cmH_2_O since SARs in this cohort did not display tonic activity (i.e., no activity at P_TR_ = 0 cmH_2_O) and several fibers routinely ceased their AP *f* as P_TR_ approached 20 cmH_2_O (4/8 fibers). The latter phenomenon generally presented in SARs with a very high overall AP *f* (e.g., 357 AP/s at 16 cmH_2_O for an SAR with a lower than typical steady state P_TR_ vs. ~ 150 AP/s mean AP *f* across all fibers at 16 cmH_2_O). Over the range of 5–15 cmH_2_O, pretreatment with Tropisetron decreased mean AP *f* compared with controls (*p* < 0.01; Figure 8). There was a significant relationship between P_TR_ and AP *f* in both naïve and pretreated fibers (*p* < 0.001) and there was a significant interaction between naïve vs. Tropisetron condition and P_TR_ (*p* < 0.05; all 2-way RM ANOVA, ranked data).

Pretreatment with Suramin was assessed between 5 and 20 cmH_2_O, and one receptor (m090714) was excluded as an outlier from percent change analysis due to its extremely high overall AP *f* and its reduction in AP/s at P_TR_ > 15 cmH_2_O (>2 Std Dev from mean across all pressures; e.g., −56% at P_TR_ = 20 cmH_2_O, vs. approximately −10% at P_TR_ = 20 cmH_2_O for all other fibers). Suramin decreased mean AP *f* compared with controls (*p* < 0.01; Figure 8). There was a significant relationship between P_TR_ and AP *f* in both naïve and Suramin (*p* < 0.001), as well as an interactive effect between naïve vs. Suramin condition and P_TR_ (*p* < 0.05; all Figure 8, 2-way ANOVA, ranked data).

Small but statistically significant (ANOVA) overall percent changes in AP *f* relative to naïve control values were observed in all test conditions, with hypoxia increasing AP *f* (F_I_O_2_ = 0.1: +9.3 ± 1.9%, *p* < 0.001, *R* = 0.9907) and hypercarbia, Tropisetron, and Suramin decreasing AP *f* (F_I_CO_2_ = 0.1: −6.2 ± 0.9%, *p* < 0.001, *R* = 0.9064; Tropisetron: −7.6 ± 1.8%, *p* = 0.005, *R* = 0.9664; Suramin: −10.6 ± 1.5%, *p* = 0.01, *R* = 0.9126).

## Discussion

We report herein: (1) the first in-depth characterization of murine SAR mechanosensitivity relative to trans-respiratory pressure in the C57BL/6 strain of mice commonly employed in biomedical research and (2) characterization of murine SAR chemosensitivity and pharmacology. Murine SARs exhibited high sensitivity with respect to action potential frequency relative to P_TR_ (i.e., action potential frequency, AP *f*), which may be important for detection of operating lung volumes relative to TLC (Vinegar et al., 1979) and robust signaling of inflation pressures within the murine tidal breathing P_TR_ range to provide appropriate signaling for the high B*_f_* of the mouse. As with previous studies on SAR mechanosensitivity, we did not find SARs to be meaningfully impacted by exposure to classic NEB stimuli (hypoxia or hypercarbia; Whitteridge and Bülbring, 1944; Bradley et al., 1976; Kunz et al., 1976) or altered by NEB-implicated signaling pathways (serotonergic or purinergic). In contrast to the very large discharge range of SAR activity in response to mechanical stimulation (inflation from FRC to TLC), we report physiologically modest, though statistically significant, influences of pharmacologic or chemical factors on SAR AP *f*. The magnitude of the latter does not support a direct physiologic link between myelinated SAR mechanoreceptors and NEBs, although our findings do not preclude either a distinct or perhaps modulatory role for NEBs during normal physiologic conditions and SAR function.

### Murine Slowly Adapting Receptors: A Specialized Vagal Afferent Population

Murine SARs displayed a 376% increase in AP *f* between FRC (P_TR_ = 0 cmH_2_O) and TLC (P_TR_ = 20 cmH_2_O; Figure 6). Overall, this relationship of increasing SAR AP *f* in response to increasing inflation pressure is consistent with current dogma surrounding SAR behavior (Sant’Ambrogio, 1982; Widdicombe, 2001). However, the magnitude (i.e., sensitivity) of this murine response is unique. Specifically, the maximum frequency at TLC of murine SARs was a remarkable ~60% greater than reported for larger mammals, such as rabbits, cat, and dogs [236.1 vs. <150 AP/s; (Widdicombe, 1954a; Miserocchi and Sant’Ambrogio, 1974; Sant’Ambrogio et al., 1974; Bartlett and St. John, 1979)]. The higher mouse AP *f* mirrors patterns in avian species of varying sizes, where smaller birds display greater SAR AP *f* and adaptation, albeit to CO_2_, rather than mechanical stimuli (Frappell et al., 2001; Hempleman et al., 2005). This supports the concept that the higher B*_f_* of small animals is accompanied by appropriately tuned sensory mechanisms, especially for breathing. This size dependence for various biological rates is echoed across biology (e.g., heart rate and gait) and analyzed through allometric scaling models (Frappell et al., 2001; Hempleman et al., 2005). The diversity of SAR responsiveness across differentially sized species is uniformly constrained below the presumed maximal discharge frequency for all species (~300 AP/s), which reflects the biophysical limitations of ion channel function (Hille, 1992). While the molecular mechanisms underpinning SAR behavior are unknown, recent reports of the essential role of the Piezo2 ion channel in mediating pulmonary mechanotransduction (e.g., as reflected by the Hering-Breuer reflex) suggests one potential candidate (Goridis, 2017; Nonomura et al., 2017; Zhong et al., 2018). Interestingly, pulmonary Piezo2 has been reported to be uniquely expressed in NEBs and vagal/spinal sensory neurons (Nonomura et al., 2017). Further investigation is required to determine the precise patterns of localization of these sensory neurons and their endings to determine whether or how Piezo2 might confer the mechanosensitivity attributed to SARs.

An early published report of murine SAR behavior examined AP *f* relative to inflation volume, an indirect SAR stimulus, in OF1 and C3H/HeJ mice (Burnet and Hilaire, 1999), whereas a later study examined C57BL/6 SAR activity during rapid (dynamic) inflation to specific target pressures in a preparation with an open chest cavity (Zhang et al., 2006). While Burnet and Hilaire only reported AP *f* in the normal tidal volume range and during tracheal occlusion (Burnet and Hilaire, 1999), Zhang et al. utilized an airway pressure protocol where inflation was initiated from 0 cmH_2_O, where many airways would be presumed to be closed (due to the open chest condition), to a target pressure of ~10 cmH_2_O, which exceeds a normal tidal breathing pressure range (Zhang et al., 2006). Zhang et al. (2006) reported data for *n* = 44 SAR at constant airway pressures of 0, 10, 20, and 30 cmH_2_O, whereas we report herein data for *n* = 85 SARs alongside histograms of their distribution of responses at pressures from 0 to 20 cmH_2_O.

In OF1 and C3H/HeJ mice, tidal volume range firing frequencies of approximately 80–100 AP/s were reported (Burnet and Hilaire, 1999), which fall within the range of AP *f* we observed herein in C57BL/6 mice inflated to P_TR_ = 5–10 cmH_2_O (note: our tidal ventilation was set to an end-inspiratory pressure of 7–8 cmH_2_O). However, unlike the former work, we have characterized the activity of SARs across the entire range of lung volumes from FRC through to TLC. Further, trans-respiratory pressure is a more appropriate stimulus for SARs as opposed to volume, which may be associated with different discharge frequencies dependent on changes in lung compliance (Sant’Ambrogio, 1982). Our study controlled for compliance using a strict volume history inflation protocol. We used P_TR_ as a surrogate for transpulmonary pressure as the mouse chest wall has a high compliance (O’Neil and Raub, 1984; Mortola, 1987; Lai and Chou, 2000) and it removes the necessity and impact of surgical opening of the chest wall.

Zhang et al. (2006) reported AP *f* of approximately 80 AP/s at an inflation pressure of 20 cmH_2_O. This is lower than our finding of 173 AP/s at 20 cmH_2_O. This difference may be related to underlying differences within the two protocols employed. Zhang et al. utilized rapid (i.e., dynamic) inflations from 0 cmH_2_O to the target pressure (i.e., 10 or 20 cmH_2_O), while we used a slow, quasi-static inflation to achieve our target pressure. However, the Zhang et al. protocol might be expected to enhance peak discharge rates rather than reduced them. While both groups employed mechanical ventilation, we used an end-inspiratory tidal pressure of 7–8 cmH_2_O and B*_f_* of 120 breaths/min, versus Zhang et al.’s pressure at the airway opening of 10 cmH_2_O and B*_f_* of 90 breaths/min. During naïve conditions, our mice were ventilated with 40% O_2_ to ensure hemoglobin saturation, versus ventilation with room air by Zhang et al. Whether the major surgery of opening the chest wall and mechanical ventilation from lung collapse obtained at 0 cmH_2_O in the open chest condition affected the underlying physiology of the SARs remains to be determined. Our preparation included minimal surgery, an intact chest walls to help preserve functional residual capacity, and mechanical ventilation settings and supplemental oxygen designed to preserve oxygenation. In contrast, Zhang et al. utilized an open chest wall to facilitate punctate identification of receptor fields as well ventilation settings, which although useful to probe receptor location, may not preserve lung volume above closing volumes or normal systemic physiology.

We observed few fibers “tonically” active at P_TR_ = 0 cmH_2_O (FRC), although all fibers were active by P_TR_ = 5 cmH_2_O. This aligns with previous descriptions of both tonic and high-threshold SARs (Yu, 2000; Zhang et al., 2006), which also reported relatively smaller representation of tonic SARs in other species [e.g., rabbit (Yu, 2000)]. SAR threshold was previously thought to reflect morphology or end-unit location, with high-threshold receptors believed to be located in the peripheral lung and low threshold (i.e., tonic) receptors in the larger, central airways (Sant’Ambrogio, 1982). While a putative localization of tonic fibers to large/extrapulmonary airways coupled with relatively few extra thoracic receptors [<20% in dogs (Bartlett et al., 1976a)] could explain our observation of few tonic receptors, the paradigm of geographically distinct SARs has been questioned by findings of both high-threshold and tonic SARs with no striking morphological distinctions in the peripheral rabbit lung (Yu et al., 2003). Thus, the underlying mechanism(s) conferring low threshold remain unclear (Mazzone and Undem, 2016), although we report for the first time the distribution of receptor threshold and frequencies reminiscent of a normal distribution.

The vast majority of murine SARs we investigated displayed a classically slow, negligible, or incomplete adaptation to static/sustained stimuli, as per previous findings (Adrian, 1933; Knowlton and Larrabee, 1946; Widdicombe, 1954a,b; Bartlett et al., 1976b; Sant’Ambrogio, 1982). Quasi-static inflation maneuvers conferred minimal to no adaptation; however, a small subset of receptors displayed intermittent or complete failure of AP *f* discharge at P_TR_ = 20 cmH_2_O or close to TLC volumes (this group was excluded from analysis). This sudden cessation/failure of activity is unlikely to be indicative of partial or complete adaptation to very high pressure, despite displaying otherwise typical SAR behavior (i.e., in the tidal breathing range) and most likely represents the so-called “Wedensky effect”–where select SARs reach unsustainably high AP *f* levels at P_TR_ approaching TLC (Adrian, 1933). The “Wedensky Effect” has previously been reported in the opossum (Farber et al., 1983) and rabbit (Guardiola et al., 2007) as occurring in SARs with very high AP *f* (Farber et al., 1983; Guardiola et al., 2007), and involves complete/partial cessation of AP *f* likely due to physiologically unsustainable over-excitation, rather than inhibition, of the nerve (Winner et al., 2005).

### SAR Mechanosensation and the Link to NEBs

The action potential discharge characteristics of pulmonary SARs are a critical sensory mechanism responsible for the Hering-Breuer reflex and control of airway smooth muscle tone (Richardson et al., 1984; Schelegle and Green, 2001). Recently, the Piezo2 protein has been reported to be a key molecular substrate responsible for mechanotransduction in the lung and has been directly implicated in the Hering-Breuer reflex (Nonomura et al., 2017). Remarkably, Nonomura et al. also reported that NEBs were the sole cell type within the lung to express Piezo2, suggesting a possible link between NEBs and mechanosensation of the Hering-Breuer reflex. Earlier work by Adriaensen’s group proposed that NEB-associated SMARs (smooth muscle-associated receptors) were the morphological counterpart of electrophysiologically characterized SARs (Adriaensen et al., 2006; Brouns et al., 2006a,b). As a result, we used neurophysiologic recordings of single SARs to address the hypothesis that NEB function is linked to SAR (i.e., SMAR) function.

Based on both aforementioned lines of inquiry (Adriaensen and Nonomura), one might expect interventions that alter NEB function to modulate the mechanosensitivity of SARs. However, chemosensory and pharmacologic interventions (Figure 3) caused changes in AP *f* of a magnitude that reflect very minimal changes in mechanical responsiveness *in vivo*, which raises the question of whether they are physiologically relevant (see Figures 5, 8). Based on the present data, we speculate that a direct physiologic link between mechanosensitive SARs and polymodal NEBs appears somewhat tenuous. This is in sharp contrast to the reported profound impact of loss of Piezo2 on the Hering-Breuer reflex and compound vagus nerve activity comprised of myelinated and unmyelinated axons (Nonomura et al., 2017). It remains to be seen whether the loss of Piezo2 function ablates or significantly reduces SAR mechanosensitivity (Nonomura et al., 2017), due to a previously unappreciated localization of Piezo 2 on SARs. Our range of interventions, which induced very minor changes when compared to the large inherent range of SAR mechanosensitivity, targeted functions of NEBs that do not appear to influence or modulate the primary role of Piezo2 on SAR neurophysiology.

Hypoxia, a key stimulus of NEBs, caused only a minor increase in SAR sensitivity (max. change: +9.3 ± 1.9%) and a minor decrease in P_TR_ threshold (−0.6 cmH_2_O). This is consistent with earlier findings that hypoxia has minimal impact on SAR activity in denervated airways (Adrian, 1933; Whitteridge and Bülbring, 1944) and modest impact in the partially innervated trachea when secondary to chemoreflex bronchoconstriction (Fisher et al., 1983; Lembrechts et al., 2011). Such changes pale in comparison with the impact of hypoxia on K^+^ current in NEBs (approximately −47%; Fu et al., 2002, 2007), with the role of NEB innervation in the NEB hypoxic *in vivo* response in rabbits (Lauweryns and Van Lommel, 1986), and with the approximately 2-fold increase in carotid sinus nerve AP *f* during exposure to 12 vs. 100% O_2_ (Kline et al., 2002).

Hypercarbia decreased SAR sensitivity slightly (max. change: −5.8 ± 1.1%) while raising P_TR_ threshold slightly (+0.5 cmH_2_O), consistent with several studies reporting small and variably inhibitory effects of CO_2_ on SARs (Mustafa and Purves, 1972; Schoener and Frankel, 1972; Sant’Ambrogio et al., 1974; Bradley et al., 1976; Kunz et al., 1976). SAR CO_2_ responsiveness may depend on location (Bartlett and Sant’Ambrogio, 1976), with CO_2_ responsiveness thought to be partially dependent on indirect activation of SARs by CO_2_ through the action of CO_2_ on neighboring cells (Bartlett and Sant’Ambrogio, 1976) or airway tone (Bartoli et al., 1974; Ingram, 1975; Bartlett and Sant’Ambrogio, 1976). In this paradigm, CO_2_-sensitive NEBs contacted by SARs would provide an attractive candidate CO_2_-sensing arrangement; however, our data do not support such a direct link, based on both the modest response we observed, and its inhibitory nature versus the excitatory impact of CO_2_ on NEBs (Livermore et al., 2015). It is worth mentioning that our hypercarbic trials occurred concurrent with hyperoxia (90% O_2_ balance) to prevent any potential hypoxic respiratory stimulus during these trials. While the effects of chronic hyperoxia on airway function and morphology are relatively well characterized (Szarek, 1989; Hershenson et al., 1992), our exposure (in the order of minutes) falls short of the hours-to-days required to observe edematous or mechanical changes to the airways that might alter SAR firing secondary to lung mechanics during hyperoxic exposure (Caldwell et al., 1966; Fisher et al., 1968; Dewar et al., 1972; Murchie et al., 1993).

Tropisetron (5-HT_3_R antagonist) and Suramin (P2R antagonist) each caused small decreases in SAR AP *f* (<10%) with only Tropisetron causing a minor but statistically significant (0.5 cmH_2_O) increase in P_TR_ threshold. This contrasts with evidence for an important role for 5-HTRs in respiration (Yoshioka et al., 1992) and of their expression on vagal nodose ganglia (Rosenberg et al., 1997) and NEBs (Fu et al., 2001), as well as of P2X_2_ and P2X_3_ receptor expression on NEBs (Fu et al., 2004). Studies in non-pulmonary hollow organs have shown that P2X_2_/P2X_3_ heterodimer expression is associated with both mechanosensory and nociceptive signal transduction (Burnstock, 2007a,b). As NEBs release ATP in response to stimulation, this ATP may activate their P2R-expressing sensory innervation (De Proost et al., 2008); however, we only observed a minimal impact of P2R blockade on SAR activity. Recent evidence suggests that 5-HTR and P2R are expressed on unmyelinated pulmonary C fibers (Lin et al., 2012; Hsu et al., 2019), emphasizing the need for further work to elucidate potential linkages between NEBs and various vagal afferent populations. While Tropisetron and Suramin can exert dose-dependent systemic effects, these are either not of direct concern to the present study of pulmonary SARs [e.g., include relaxation of gut smooth muscle, inhibition of spontaneous stomach smooth muscle contraction, decreases in cardiac output, blood pressure, or heart rate (Den Hertog et al., 1989; Hof et al., 1993; Xue et al., 1998; Montaño et al., 2011)] or are minimized at the doses chosen herein.

Whether respiratory stimuli, such as mechanical stretch, act directly on NEBs, their innervation, or a combination of the two *in vivo,* remains unknown (Brouns et al., 2009). The low observed effect of Suramin pretreatment on recorded AP *f* could also represent an inadvertent sampling bias, as P2R expression only occurs on approximately one quarter of murine NEB-innervating vagal fibers, making it methodologically challenging to select for such a population during vagal dissection without being able to characterize its behavior or refer to a signature profile of activation (Brouns et al., 2009). Interestingly, recent work has demonstrated expression of another P2R (P2ry1) on vagal fibers innervating NEBs, with Piezo2 co-expressed on 44% of P2ry1-expressing neurons (Chang et al., 2015). Optogenetic stimulation of P2ry1-expressing neurons results in a cessation of inhalation and prolongation of exhalation (Chang et al., 2015), though the impact of P2ry1 at the level of the individual afferent has not yet been characterized. Generally, the small impacts of both 5-HT_3_R and P2R blockade reported herein argue for a modest role for NEBs in SAR signaling, while not excluding the possibility of these receptors playing a modulatory role under other conditions. Future studies addressing the impact of chemosensory challenge or 5-HT_3_R and P2R blockade on other vagal afferent populations (e.g., rapidly adapting receptors; C fibers), as well as the combined impact of chemosensory challenge in the presence of pharmacologic blockade, are needed to further elucidate the potential role of NEBs in modulating vagal afferent activity.

## Conclusion

Characterization of the mouse pulmonary Slowly Adapting Receptor (SAR) sensory discharge over the FRC to TLC range (0–20 cmH_2_O P_TR_) revealed receptors with low P_TR_ thresholds of activation, high discharge frequencies, and high mechanosensitivity compared with those observed in larger species. Low pressure activation thresholds and high frequencies likely provide important feedback on operating lung volumes that reflect inspiratory capacity, which is a key variable in hyperinflation-induced dyspnea (O’Donnell et al., 2016, 2017). Our data support the neurophysiologic tuning of the magnitude of afferent discharge to the relatively high breathing rate of small mammals, such as the mouse, with the need for enhanced responsiveness to provide temporally aligned feedback to the brainstem. Chemosensory (hypoxia and hypercarbia) and pharmacologic (serotonergic and purinergic blockade) interventions chosen due to their documented and significant impact on neuroepithelial body (NEB) activation caused only minor differences in SAR activity. The low magnitude of impact of these interventions, compared with the dramatic alteration of SAR activity in response to mechanical stretch, does not immediately support recent hypotheses of a significant role for chemosensation by SARs or a direct link of SARs to polymodal NEBs. Rather, our findings would suggest, at best, a modest modulatory role for 5-HT_3_Rs or P2Rs in SAR signaling or a possible role for NEBs in modulating chemosensory function (e.g., high-threshold Aδ receptors, C fibers, or sympathetic afferents), as supported by NEB production and release of chemosensor-activating mediators. In concert, our findings point to the need for additional studies to address SAR function and morphology *in vivo*. This is especially true with respect to the Piezo2 mechanoreceptor candidate and with respect to diverse populations of vagal afferents, such as NEBS.

## Data Availability Statement

The raw data supporting the conclusions of this article will be made available by the authors, without undue reservation.

## Ethics Statement

The animal study was reviewed and approved by the Queen’s University Animal Care Committee in compliance with the Canadian Council of Animal Care.

## Author Contributions

All authors contributed significantly to the design of the study, data interpretation, editing of the manuscript and to the article and approved the submitted version. NJD and SGV contributed significantly to data acquisition. NJD contributed significantly to data analysis and wrote the initial draft.

## Funding

NJD was supported by an NSERC Post-Graduate Scholarship. JTF was supported by CIHR MOP-81211.

## Conflict of Interest

The authors declare that the research was conducted in the absence of any commercial or financial relationships that could be construed as a potential conflict of interest.

## Publisher’s Note

All claims expressed in this article are solely those of the authors and do not necessarily represent those of their affiliated organizations, or those of the publisher, the editors and the reviewers. Any product that may be evaluated in this article, or claim that may be made by its manufacturer, is not guaranteed or endorsed by the publisher.

## Abbreviations

AP: Action potential
AP *f*: Action potential discharge frequency
AP/s: Units for action potential discharge frequency, i.e., action potentials per second
ASM: Airway smooth muscle
B*_f_*: Breathing frequency
NOX2: NADPH oxidase 2
NEBs: Neuroepithelial bodies
PNECs: Pulmonary neuroendocrine cells
P2R: Purinergic (type 2)
RARs: Rapidly adapting receptors
SARs: Slowly adapting receptors
SMARs: Smooth muscle-associated receptors
P_TR_: Trans-respiratory pressure
Threshold: P_TR_ threshold for activation
5-HT: 5-Hydroxytryptamine, serotonin
5-HT_3_R: 5-HT (type 3) receptor
